# Supplementary material for: MetaQTL: a package of new computational methods for the meta-analysis of QTL mapping experiments
Source: BMC Bioinformatics. 2007 Feb 8;8:49. doi: 10.1186/1471-2105-8-49 (PMC1808479; doi:10.1186/1471-2105-8-49)
Supplement: Additional File 7 — MetaQTL Package : jar file and tutorial. This Zip archive contains both the MetaQTL JAR file and the files of the tutorial. [file 1471-2105-8-49-S7.zip › org.inra.metaqtl/doc/org/metaqtl/bio/entity/adapter/BioEntityAdapter.html]

BioEntityAdapter


|  |  |  |
| --- | --- | --- |
| **Overview** \| **Package** \| **Class** \| **Use** \| **Tree** \| **Deprecated** \| **Index** \| **Help** | |  |
| PREV CLASS   **NEXT CLASS** | **FRAMES**    **NO FRAMES**     **All Classes** |  |
| SUMMARY: NESTED \| FIELD \| CONSTR \| METHOD | DETAIL: FIELD \| CONSTR \| METHOD |  |


---


## org.metaqtl.bio.entity.adapter Class BioEntityAdapter

```
java.lang.Object
  org.metaqtl.bio.entity.adapter.BioEntityAdapter
```

**All Implemented Interfaces:**: IBioAdapter

**Direct Known Subclasses:**: BioEntityCollectionAdapter, GeneticMapBeanAdapter, LGroupBeanAdapter, LocusBeanAdapter, OntologyBeanAdapter, OntologyTermBeanAdapter

---

``` public abstract class BioEntityAdapter extends java.lang.Object implements IBioAdapter ```

Class Description Here

**Author:**
:   Jean-Baptiste Veyrieras

---

**Constructor Summary**

`BioEntityAdapter()`


| **Method Summary** | |
| --- | --- |
| `static void` | `fromEntity(IBioEntity entity, BioEntityBean bean)` |
| `static void` | `toEntity(BioEntityBean bean, IBioEntity entity)` |

**Methods inherited from class java.lang.Object**

`clone, equals, finalize, getClass, hashCode, notify, notifyAll, toString, wait, wait, wait`

**Methods inherited from interface org.metaqtl.bio.IBioAdapter**

`fromEntity, toEntity`

**Constructor Detail**

### BioEntityAdapter

```
public BioEntityAdapter()
```


**Method Detail**

### toEntity

```
public static void toEntity(BioEntityBean bean,
                            IBioEntity entity)
```

---


### fromEntity

```
public static void fromEntity(IBioEntity entity,
                              BioEntityBean bean)
```


---


|  |  |  |
| --- | --- | --- |
| **Overview** \| **Package** \| **Class** \| **Use** \| **Tree** \| **Deprecated** \| **Index** \| **Help** | |  |
| PREV CLASS   **NEXT CLASS** | **FRAMES**    **NO FRAMES**     **All Classes** |  |
| SUMMARY: NESTED \| FIELD \| CONSTR \| METHOD | DETAIL: FIELD \| CONSTR \| METHOD |  |


---
